# Molecular mechanisms of cold pain

**DOI:** 10.1016/j.ynpai.2020.100044

**Published:** 2020-01-28

**Authors:** Donald Iain MacDonald, John N. Wood, Edward C. Emery

**Affiliations:** Wolfson Institute of Biomedical Research, UCL, United Kingdom

**Keywords:** Pain, Ion channels, Thermosensation, Nociception, Cold, Cold allodynia, Neuropathic pain, Dorsal root ganglion

## Abstract

The sensation of cooling is essential for survival. Extreme cold is a noxious stimulus that drives protective behaviour and that we thus perceive as pain. However, chronic pain patients suffering from cold allodynia paradoxically experience innocuous cooling as excruciating pain. Peripheral sensory neurons that detect decreasing temperature express numerous cold-sensitive and voltage-gated ion channels that govern their response to cooling in health and disease. In this review, we discuss how these ion channels control the sense of cooling and cold pain under physiological conditions, before focusing on the molecular mechanisms by which ion channels can trigger pathological cold pain. With the ever-rising number of patients burdened by chronic pain, we end by highlighting the pressing need to define the cells and molecules involved in cold allodynia and so identify new, rational drug targets for the analgesic treatment of cold pain.

## Highlights

• The sensation of cooling is essential for survival.
• Ion channels expressed in peripheral cold-sensing neurons transduce cold stimuli.
• Sodium and potassium channels control terminal excitability at low temperatures.
• Chronic pain patients with cold allodynia experience mild cooling as pain.
• Changes in sensory neuron excitability drive cold allodynia.

## 1. Introduction

The sensation of cooling is essential for survival, with animals evolving multiple strategies to mitigate, avoid and escape low temperatures. Cold sensing depends on peripheral input from specialized sensory neurons that detect drops in temperature through cutaneous nerve endings. In recent years, the scientific study of cold sensation has been revolutionized by the discovery and characterization of cold transducer molecules – ion channels expressed in sensory neurons that are gated by decreasing temperature (Foulkes and Wood, 2007). Activation of these ion channels by cooling leads to membrane depolarization, action potential firing, and ultimately the perception of cold by the nervous system (Palkar et al., 2015).

**Fig. 1:**
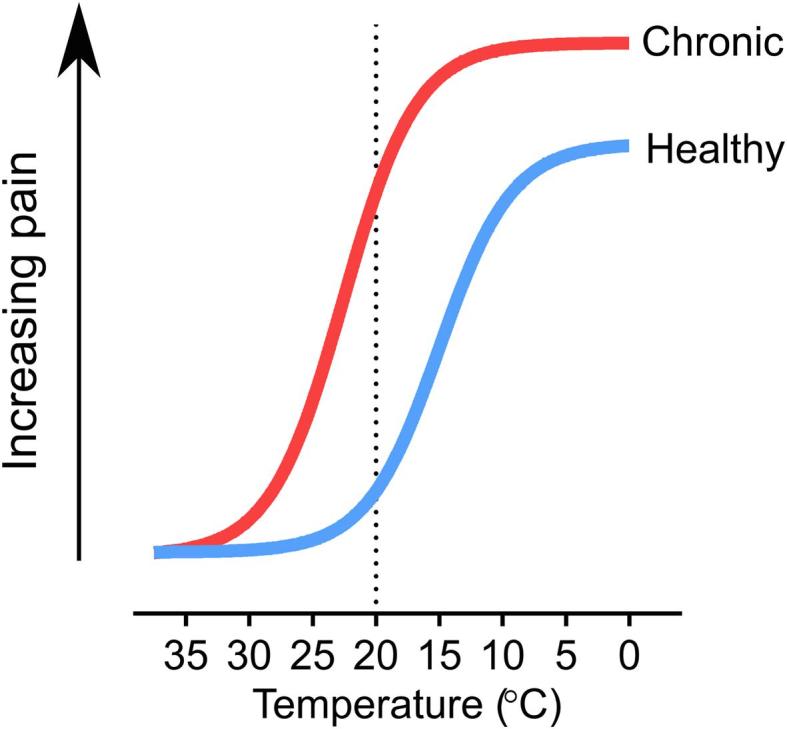
Schematic of human cold pain sensitivity in health and disease. In chronic pain conditions, the sensitivity to decreasing temperature is increased, which typically manifests as an increase in cold-induced pain. The dashed line represents the approximate threshold at which cooling begins to evoke noticeable pain in healthy individuals.

**Fig. 2:**
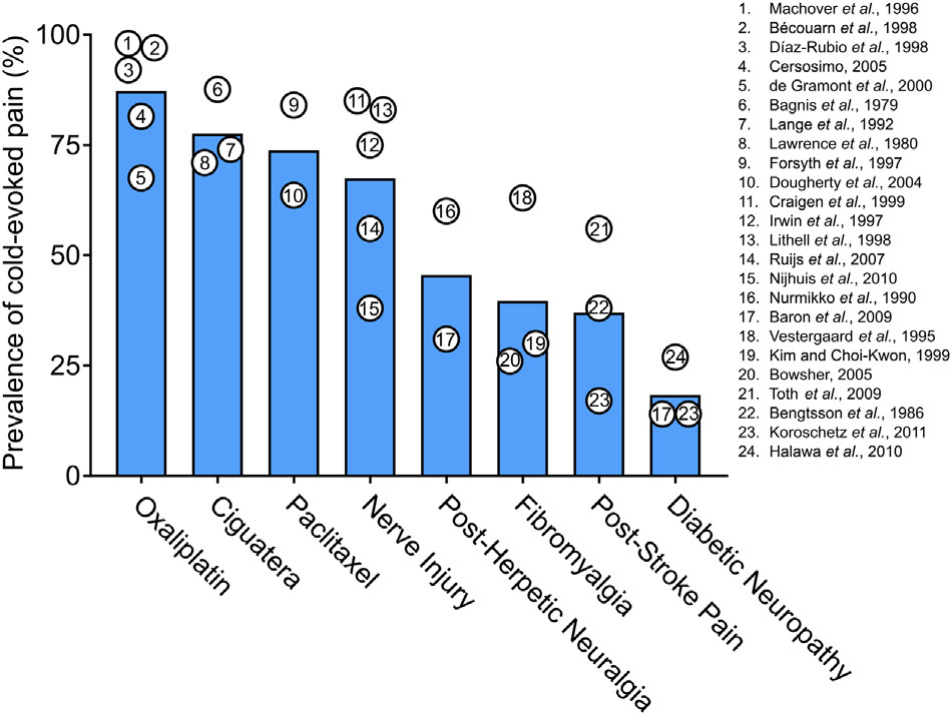
Average estimated prevalence of cold-evoked pain in different human pain states. Data points represent the prevalence of pain evoked by cooling or cold stimuli reported among chronic pain patients in individual clinical studies (Baron et al., 2009, Bécouarn et al., 1998, Bengtsson et al., 1986, Bowsher, 2005, Cersosimo, 2005, Craigen et al., 1999, de Gramont et al., 2000, Díaz-Rubio et al., 1998, Dougherty et al., 2004, Forsyth et al., 1997, Halawa et al., 2010, Irwin et al., 1997, Kim and Choi-Kwon, 1999, Koroschetz et al., 2011, Lange et al., 1992, Laugier et al., 1979, Lawrence et al., 1980, Lithell et al., 1998, Machover et al., 1996, Nijhuis et al., 2010, Nurmikko et al., 1990, Ruijs et al., 2007, Toth et al., 2009, Vestergaard et al., 1995).

Extreme cold is experienced as pain because cold is a noxious stimulus that causes profound, irreversible tissue damage at temperatures above and below freezing (Viana and Voets, 2019). In healthy individuals, the temperature at which skin cooling begins to evoke pain and subsequent protective behaviour is about 20 °C, minimizing exposure to dangerously cold stimuli. Among the most unpleasant symptoms of people suffering from chronic pain, however, is cold allodynia, when cooling to normally innocuous temperatures is experienced as excruciating pain (Fig. 1). Chronic pain afflicts a fifth of people worldwide, with many patients refractory to treatment (Breivik et al., 2006). Of these patients, cold allodynia is a common complaint of people suffering from neuropathic pain triggered by chemotherapy, ciguatera poisoning, peripheral nerve injury and post-herpetic neuralgia (Fig. 2) (Jensen and Finnerup, 2014, Yin et al., 2015).

Cold allodynia is a maladaptive and inappropriate response of the nociceptive sensory system to mild cooling. Despite advances in identifying cold-gated ion channels, the molecular mechanisms driving pathological cold-evoked pain are unclear (Lolignier et al., 2016). To develop better treatments for chronic pain, it is imperative we understand cold sensation at the molecular level in both health and disease. Here we review the ion channels that detect cooling and extreme cold, discuss their contribution to cold allodynia and end by highlighting their potential as therapeutic targets for treating chronic pain.

## 2 Sensory biology of cooling and cold pain

Peripheral sensory neurons have cell bodies in the dorsal root and trigeminal ganglia and conduct action potentials via pseudo-unipolar axons from peripheral tissues to the central nervous system in response to noxious and innocuous stimuli. Although thermosensation is not as extensively studied as touch or nociception in humans, afferents in glabrous skin that respond to cooling have been identified by microneurography (Campero et al., 2009). Cold-responsive nerves were first recorded in classical electrophysiological experiments carried out in cats and monkeys (Dubner et al., 1975, Zotterman, 1936). The dorsal root ganglia neurons that detect cooling project mainly to the superficial laminae of the dorsal horn: layers I, II and III. Here incoming sensory signals drive reflex arcs and are molded by spinal cord interneuron networks before being transmitted to the brain to drive appropriate behavioural responses.

Cold-sensing afferents encompass a menagerie of different poly- and unimodal, small and large fibre types (Yin et al., 2015). Low-threshold thermoreceptors respond to mild cooling, while high-threshold cold nociceptors are activated by extreme cold. C and A-delta low-threshold thermoceptors are spontaneously active at neutral skin temperatures, but their firing frequency increases in response to small temperature drops, and rapidly adapts once steady-state temperature is reached. In contrast, the high-threshold cold nociceptors are normally quiescent, but show prolonged, lower frequency and delayed firing after extended cooling into the noxious temperature range. While low-threshold cold thermoreceptors are usually unimodal, the cold nociceptors also fire in response to noxious heat and mechanical stimuli. Lastly, temperatures below 0 °C cause all normally cold-insensitive nociceptors to fire, presumably via freezing-induced tissue damage (Simone and Kajander, 1996).

The psychophysical threshold for cooling perception in healthy humans is a drop of just 1–2 °C, with cold pain perceived below about 20 °C, and a distinct stinging cold sensation felt at subzero temperatures (Jensen and Finnerup, 2014). Despite inter-individual variability, these values generally do map onto sensory afferent cold thresholds. Interestingly, mice have a similar detection threshold for cooling of ~2 °C (Milenkovic et al., 2014). Compared to other stimulus modalities, cold sensation is trickier to study in mice. The Cold Plate test, for example, measures the time to hindpaw lifting of animals placed on a cooled surface. Latencies reported in the literature range from 5 to 200 s for a plate held at 0 °C, as mice adopt a pose that minimizes exposure to the cold (Mckemy, 2010). An understanding of both the ethological validity and translational relevance of these tests is crucial to interpreting mouse behavioural phenotypes arising from genetic perturbations of cells and molecules controlling cold sensation. For convenience, we summarize in Table 1 the impact of genetically manipulating a range of ion channels on selected assays of acute cold sensitivity.

**Table 1:** Acute cold sensitivity of ion channel KO and cell ablated mice. The symbol ‘↓’ denotes reduced cold sensitivity, ‘↑’ denotes cold hypersensitivity and ‘–’ denotes unchanged cold sensitivity on different cold behavioural assays following genetic manipulation of ion channels and sensory neuron subsets.

Mouse Line			Behavioural Assay			
Type	Channel (s)	Manipulation	Dry Ice	Acetone	Thermal Preference	Cold Plate
Trp	*Trpm8*	KO	↓(Morenilla-Palao et al., 2014, Pogorzala et al., 2013)	↓(Bautista et al., 2007, Dhaka et al., 2007, Knowlton et al., 2013)	↓(Bautista et al., 2007, Dhaka et al., 2007, Knowlton et al., 2013, Knowlton et al., 2010, Pogorzala et al., 2013)	↓(Bautista et al., 2007, Colburn et al., 2007, Gentry et al., 2010, Knowlton et al., 2013), – (Dhaka et al., 2007)
		DTR	↓(Pogorzala et al., 2013)	↓(Knowlton et al., 2013)	↓(Knowlton et al., 2013, Pogorzala et al., 2013)	↓(Knowlton et al., 2013)
	*Trpa1*	KO	–(Morenilla-Palao et al., 2014)	↓(Kwan et al., 2006), – (Bautista et al., 2006)		↓(Gentry et al., 2010, Karashima et al., 2009, Kwan et al., 2006), – (Bautista et al., 2006)
		DTR			–(Yarmolinsky et al., 2016)	
	*Trpa1/Trpm8*	Double KO			↓(Knowlton et al., 2010, Winter et al., 2017)	
	*Trpc5*	KO			–(Zimmermann et al., 2011)	–(Zimmermann et al., 2011)

K2P	*TRAAK*	KO			–(Noël et al., 2009)	– (Noël et al., 2009)
	*TREK1*	KO		–(Alloui et al., 2006)		
	*TRAAK/TREK1*	Double KO			↑(Noël et al., 2009)	↑(Noël et al., 2009)
	*TREK2*	KO			↑(Pereira et al., 2014)	–(Pereira et al., 2014)
	*TRAAK/TREK1/TREK2*	Triple KO			↑(Pereira et al., 2014)	↑(Pereira et al., 2014)
	*TASK-3*	KO	↑(Morenilla-Palao et al., 2014)	↑(Morenilla-Palao et al., 2014)		
	*TRESK*	KO	↑ (Castellanos et al., 2019)		↑(Castellanos et al., 2019)	↑(Castellanos et al., 2019)

Na_V_	*Na_V_1.7*	Avil-Cre KO		↓(Minett et al., 2012)	–(Minett et al., 2012), ↓(Minett et al., 2014a, Minett et al., 2014b)	
		Wnt1-Cre KO		↓(Minett et al., 2012)	–(Minett et al., 2012)	
	*Na_V_1.8*	KO	–(Luiz et al., 2019)	–(Luiz et al., 2019, Minett et al., 2012)	↓(Minett et al., 2012)	↓(Luiz et al., 2019, Zimmermann et al., 2007)
		DTA	↑(Luiz et al., 2019)	↑(Luiz et al., 2019)	–(Minett et al., 2014a, Minett et al., 2014b)	↓(Zimmermann et al., 2007), ↑(Luiz et al., 2019)
	*Na_V_1.9*	KO			↓(Lolignier et al., 2015)	↓(Lolignier et al., 2015), –(Amaya et al., 2006)

### 2.1 Molecular transduction of cooling by Trp channels

Menthol, the active ingredient in mint, is used by toothpaste and chewing gum manufacturers to simulate freshness and cooling. This is because menthol activates and sensitizes low-threshold cold thermoreceptors, pointing to a commonality in the detection of cold and the detection of menthol-containing substances. The cloning of a gene encoding the non-selective cation channel Trpm8 – gated both by menthol and decreasing temperatures – revolutionized the field by identifying a molecular substrate for cold-sensing (McKemy et al., 2002, Peier et al., 2002). Trpm8 is expressed mainly in small diameter sensory neurons, akin to the C fibre cold thermoreceptors. Fluorescent tracing using Trpm8-eGFP knockin mice show Trpm8-positive afferents terminate centrally in layer I of the spinal cord dorsal horn and peripherally in the epidermis, where they are optimally positioned to sense changes in ambient temperature (Dhaka et al., 2008).

Global deletion of Trpm8 impairs cold sensation in mice, with reduced avoidance behaviour for temperatures between 30 °C and 15 °C on the thermal place preference test and a loss of firing in sensory afferents to low-threshold cooling stimuli (Bautista et al., 2007, Colburn et al., 2007, Dhaka et al., 2007). Trpm8 knockout (KO) animals are also unable to learn a perceptual task where mice are trained to respond to cooling of the forepaw from 32 °C to 22 °C (Milenkovic et al., 2014). Avoidance of noxious cold is, however, preserved in Trpm8 KO mice, suggesting another transducer is required for detection of high-threshold cold stimuli. When Trpm8-positive neurons are killed in adulthood by diphtheria toxin injection into Trpm8-DTR mice, cold discrimination and cooling-induced analgesia is abolished, but residual cold-sensing is present at lower temperatures (Knowlton et al., 2013, Pogorzala et al., 2013).

*In vivo* imaging of the spinal cord reveals that mild, but not noxious, cooling-evoked activity in dorsal horn neurons is diminished in Trpm8-DTR mice, confirming that Trpm8-expression defines a subset of peripheral neurons responsible for innocuous cold input (Ran et al., 2016). Imaging of trigeminal ganglia where GCaMP5 was restricted to Trpm8-positive neurons using Trpm8-Cre confirms these cells are overwhelmingly selective for oral cooling, with 90% responding to cold alone (Yarmolinsky et al., 2016). A later *in vivo* imaging study found 87% of menthol-responsive trigeminal neurons responded to cooling. Contrastingly, only 32% of all cold-sensing neurons were activated by menthol (Leijon et al., 2019). This is consistent with a study of cutaneous cold-sensitive afferents in Trpm8-EGFP mice using the *ex vivo* somatosensory preparation which preserves intact the hairy skin, saphenous nerve, dorsal root ganglia and spinal cord. Intracellular recordings of dorsal root ganglia somata revealed numerous Trpm8-negative cold afferents among all fibre classes (Jankowski et al., 2017).

What mediates Trpm8-independent cold transduction? Trpa1 is directly activated by cooling below 10 °C in recombinant systems and so could control the sensation of painful cold (Story et al., 2003). However, the channel is promiscuous and integrates numerous noxious stimuli, including force, heat, inflammatory mediators and pungent compounds (Bautista et al., 2013). Whether Trpa1 is a *bona-fide* cold sensor *in vivo* remains contentious. Two studies of Trpa1 KO mice indicate the channel is essential for nocifensive behaviours evoked by the 0 °C Cold Plate, while another found no differences across multiple, more robust cold pain behavior tests (Bautista et al., 2006, Karashima et al., 2009, Kwan et al., 2006). No cold responses, even to 1 °C stimulation, were detected by imaging of murine trigeminal ganglia expressing GCaMP in Trpa1-positive neurons (Yarmolinsky et al., 2016). A two-plate avoidance test also showed normal avoidance of 5 °C by Trpa1-DTR mice, although complete ablation of all Trpa1-expressing neurons was not confirmed in this study (Yarmolinsky et al., 2016).

### 2.2 Structure and function of a cold transducer

As the only cold transducer for which the evidence is unequivocal, Trpm8 has been studied in great detail at the molecular and structural level. Trpm8 channels form homotetramers, with cytosolic N and C terminal domains and six transmembrane domains. Early structure-function studies focused on defining the molecular basis of agonist-sensitivity. Using a high-throughput random mutagenesis screen of ~14,000 mutant Trpm8 channels, residue Y745, mapped to transmembrane segment 2, and residue L1009, in the TRP domain of the C terminal, were pinpointed as crucial for menthol, but not cold sensitivity (Bandell et al., 2006). A CryoEM structure for Trpm8 from collared flycatcher has recently been reported at ~4.1 Å – interestingly, residue Y745 was actually identified on transmembrane segment 1 facing the centre of the voltage-sensitive like domain cavity (Yin et al., 2018). CryoEM structures of Trpm8 in complex with icilin and menthol analog WS-12 have now conclusively demonstrated that the binding site for cold mimetics is within this voltage-sensitive like domain cavity (Yin et al., 2019).

Reconstitution of Trpm8 in a planar lipid bilayer results in a channel that is activated by cooling, in a manner dependent on PIP2, indicative of a direct gating by the cold (Zakharian et al., 2010). Trpm8, like most Trp channels, is weakly voltage-sensitive and bears a topographic similarity to voltage-gated potassium channels. Mutagenesis screens have identified Trpm8 mutants affecting voltage-dependence that also result in altered thermal activation and menthol-sensitivity (Voets et al., 2007). For example, substitution of R842 in the S4 and S4-5 linker domain with alanine to neutralize the positive gating charge shifted the voltage-dependence of activation to more depolarized potentials, resulting in reduced agonist and thermal sensitivity (Voets et al., 2007). Cooling and cold mimetics may therefore act via the voltage-sensor to shift the voltage-dependence of activation to more hyperpolarized potentials, through an integrative mechanism of channel gating.

A promising approach for identifying residues involved in cold sensitivity is to study orthologues of the channel. For example, hibernating rodents must withstand prolonged exposure to the winter cold. Ground squirrels and Syrian hamsters, both hibernating species, show enhanced cold tolerance on the thermal place preference test compared to non-hibernating mice. Interestingly, cold-sensing neurons from ground squirrels showed normal activation by Trpm8 agonists but reduced sensitivity to the cold. Substitution of the squirrel Trpm8 transmembrane core domain with a rat orthologue rescues cold sensitivity. By systematically replacing individual amino acids, six residues were identified in the transmembrane core that were sufficient to confer normal cold sensitivity to squirrel Trpm8 and that therefore likely control the response to temperature of rodent Trpm8 (Matos-Cruz et al., 2017).

### 2.3 Molecular control of thermoregulation

The unconscious detection of cooling drives autonomic processes that maintain core body temperature in endothermic species in the face of decreasing environmental temperature (Tan and Knight, 2018). Accumulating evidence points to Trpm8 as the critical channel controlling cold-induced thermoregulation. Menthol application causes a rise in body temperature with increased brown adipose tissue thermogenesis (Tajino et al., 2011). By contrast, treating rats and mice with Trpm8 antagonists transiently decreases body temperature as measured by implanted telemetry probes (Gavva et al., 2012). Trpm8 KO animals also exhibit decreased core body temperature when housed in the cold. Interestingly, energy homeostasis goes awry in Trpm8-deleted mice, with animals developing over-eating and obesity later in life, evidence of a mechanistic link between feeding control and thermoregulation (Reimúndez et al., 2018).

Comparative studies provide unique insights into how evolution operates at the molecular level on the Trpm8 gene to control thermoregulation across different climatic conditions. Recently, the sequencing of the woolly mammoth genome allowed for the resurrection of the mammoth Trpm8 channel which displayed intact agonist-sensitivity but effectively absent cold sensitivity, consistent with the adaptation of this extinct species to Ice Age conditions (Chigurapati et al., 2018). In humans, a single nucleotide polymorphism (SNP) variant rs10166942 ~1 kB upstream of Trpm8 shows enormous variation across the population. Allele frequencies range from 5% in Nigeria to 88% in Finland and strongly correlate with latitude and temperature in a manner indicative of strong positive selection. Interestingly, genome-wide association studies show the same variant is strongly associated with migraine, and Trpm8 modulators have been proposed to treat migraine (Dussor and Cao, 2016, Gormley et al., 2016). The SNP is predicted to play a regulatory role and may contribute to the control of Trpm8 expression, tuning thermosensation in different populations to the local climate (Key et al., 2018). That distinct molecular alterations in the Trpm8 coding gene and associated regulatory elements can be linked to gross evolutionary adaptations to the cold in humans and animals further evidences that Trpm8 is the major detector of ambient cooling and primary ‘molecular thermostat’ for cold-induced thermoregulation in mammals.

### 2.4 Ionic basis of the ‘missing sensitivity’ to cold

Cold-sensitive dorsal root ganglia neurons have been observed in culture that lack responses to menthol and AITC, and therefore probably do not express Trpm8 or Trpa1 (Munns et al., 2007). This finding is recapitulated *in vivo* – only 36% of trigeminal cold neurons responded to canonical Trp agonists in the oral cavity (Leijon et al., 2019). Both Trpa1 and Trpm8 have also been shown to be dispensable for the noxious cold sensitivity of the dental pulp (Michot et al., 2018). Interestingly, ablation of Trpv1 lineage neurons – a heterogeneous set of nociceptors and thermoreceptors – causes a reduction in noxious cold sensation significantly greater than that seen in mice lacking Trpm8 cells alone, but which cannot be ascribed to Trpa1 (Pogorzala et al., 2013). This is corroborated by the loss of spinal cord responses to strong cold in Trpv1 DTR mice (Ran et al., 2016). Clearly, there is a ‘missing sensitivity’ to noxious cold in mammalian sensory neurons that cannot be accounted for by Trpm8 or Trpa1. What alternative transducers are present in nociceptors? ENaC is directly gated by cold *in vitro* while ASICs are strongly modulated by cooling, however their role in cold-sensing has not been explicitly tested *in vivo* (Askwith et al., 2001). Recombinant Trpc5 homomers are cold-sensitive in the innocuous range but no deficit is seen in the KO animal (Zimmermann et al., 2011). A recent *C. elegans* screen identified a cold-gated metabotropic glutamate receptor GLR-3. The vertebrate homolog GluK2 is cold-sensitive and its knockdown impaired the response of mouse DRG neurons to cooling down to 10 °C, *in vitro* (Gong et al., 2019).

Closure of background potassium channels that maintain the hyperpolarized resting membrane potential is also proposed to mediate depolarization evoked by cooling (Reid and Flonta, 2001). In identified cold-responsive neurons voltage-clamped at −60 mV, a cold-induced inward current with negative reversal potential was accompanied by decreasing membrane conductance, and no increase in intracellular calcium, consistent with shutting of background potassium channels (Viana et al., 2002). Molecular identification of these potassium channels has proved challenging. The two-pore domain leak potassium channels (K2P) TREK1, TREK2 and TRAAK all undergo loss-of-function at low temperatures and are expressed in small-diameter peripheral sensory neurons (Kang et al., 2005, Patel et al., 1998, Viatchenko-Karpinski et al., 2018). Double KO of TREK1 and TRAAK results in increased numbers of cold-sensitive neurons in culture and cold-activated fibres in the skin, matched by enhanced sensitivity to noxious cold temperatures (Noël et al., 2009). TREK2-deficient mice have increased responses to innocuous cooling, but cold pain is unchanged (Pereira et al., 2014). Transcriptomic characterization of cold-sensing or Trpm8-positive neurons isolated by FACS shows an enrichment of TASK-3, a pH-sensitive K2P channel (Luiz et al., 2019, Morenilla-Palao et al., 2014). Selective block of this channel alters the thermal threshold of cold-sensing neurons, and the mouse KO shows a moderate behavioural hypersensitivity to cold (Morenilla-Palao et al., 2014).

K2P channels play at least a modulatory role in the control of the sensory neuron response to cold. Testing whether closure of a given channel is necessary for cold-induced excitation and in which neurons would require selective agonists or dynamic clamp experiments. A complex and changing complement of these channels may be present at any particular afferent terminal, in both homomeric and heteromeric forms, and whose closure can together trigger cold-induced action potentials in the absence of Trpm8 or Trpa1 (Blin et al., 2016).

### 2.5 Control of excitability at low temperatures by voltage-gated channels

Cold-sensing neurons must, by definition, reliably fire action potentials at low temperatures unfavourable to spike initiation due to cooling-induced inactivation of sodium channels. Evolution provides an elegant solution to this paradox. The sensory neuron-enriched TTX-resistant sodium channels Na_V_1.8 and Na_V_1.9 display unusual biophysical adaptations to operate during extreme cooling. Na_V_1.8 does not show cold-induced slow inactivation and so can propagate action potentials at 10 °C when all other sodium channels are inactive. Behavioural tests suggest the channel is essential for pain at low temperatures (Zimmermann et al., 2007). Consistent with the purported role of Na_V_1.8 in cold pain, Cre-dependent diphtheria toxin-mediated ablation of Na_V_1.8-positive neurons abolishes escape behavior in response to ongoing extreme cold (Abrahamsen et al., 2008). Corroborating these findings, we recently found that Na_V_1.8 deletion reduces the activity of neurons responding to prolonged cooling *in vivo*, mirrored by absent jumping when the mice are exposed to a −5 °C cold plate (Luiz et al., 2019).

Compared to Na_V_1.8, the activation threshold of Na_V_1.9 is hyperpolarized which, together with its slow inactivation, means it generates a persistent inward current at rest (Cummins et al., 1999). Functional upregulation of Na_V_1.9 in cold-responsive nociceptors thus amplifies subthreshold potentials evoked by cooling enabling prolonged spiking in the cold. Na_V_1.9 KO mice are consequently less responsive to noxious cold (Lolignier et al., 2015). A heterozygous gain-of-function missense mutation in SCN11A encoding Na_V_1.9 (p.V1184A) has been identified in a human family by whole-exome sequencing. Family members carrying the mutation experience attacks of pain in the extremities that are triggered and aggravated by cooling. Mutant channels show hyperpolarized voltage-dependence of activation and cause cold-resistant hyperexcitability when transfected into mouse nociceptors, supporting a contribution of Na_V_1.9 to nociception at low temperatures (Leipold et al., 2015).

Despite compelling evidence for a role of Na_V_1.8 and Na_V_1.9 in acute cold pain, single cell RNA sequencing of DRG neurons shows these channels have limited overlap in expression with the known cold transducer Trpm8 (Usoskin et al., 2014). Indeed, Trpm8-expressing neurons fire TTX-sensitive action potentials, with a substantial contribution from Na_V_1.1 (Griffith et al., 2019). This begs the question which sodium channel triggers the action potential in the innocuous cold detectors, if the cold-resistant isoforms are unavailable? A possible solution comes from a recent study of corneal afferents, notable for their high incidence of cold sensitivity, which showed that the action potential initiation zone is physically removed from the peripheral terminal, located further along the intraepithelial fibres (Goldstein et al., 2019). In Trpm8-positive cold sensors, sodium channels and the spike initiation zone may be sufficiently distant from the peripheral terminal to be unaffected by local cooling of the receptive field.

**Fig. 3:**
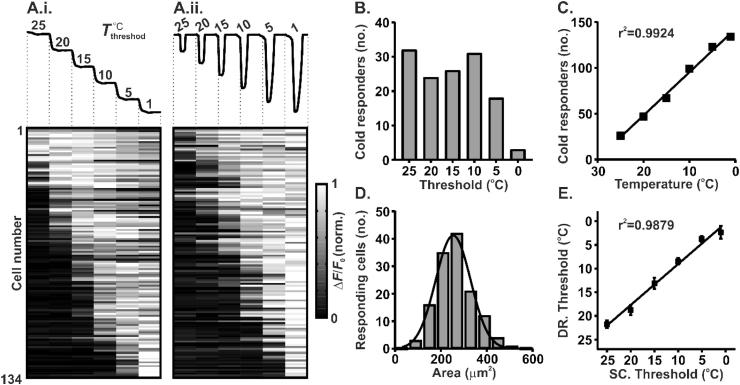
*In vivo* calcium imaging of dorsal root ganglia reveals variable thermal activation thresholds of cold-sensing neurons (Luiz et al., 2019). (A) Normalised fluorescence response from 134 cold-sensitive neurons expressing GCaMP3 following a staircased (A.i.) or drop temperature stimulus (A.ii.). The cooling protocols are shown at the top of the figure. Each row represents the response from the same neuron to each stimulus protocol. (B) Summary of the threshold of cold-sensing neuron activation observed following a staircased cooling protocol as in (A.i.). (C) Number of neurons activated by different cooling temperature drops as in (A.ii.) (linear regression: *y* =  − 4.715 ∗ *x* + 142.4). (D) Histogram of cell area for cold-sensing neurons (Least squares Gaussian; Bin width is 60 μm^2^; Mean = 253.6 μm^2^, Std. Dev. 76.06 μm^2^). (E) Relationship between mean thresholds of activation in response to a drop (DR) cooling stimulus versus a staircased (SC) cooling stimulus (linear regression: *y* = 0.8652 ∗ *x* + 0.3839). Error bars denote S.E.M.

Trpm8-expressing cold afferents exhibit enormous variability in their thermal thresholds. Using *in vivo* imaging, we have shown that cold-sensing neurons are small in size and have thresholds that tile the whole range of non-zero temperatures below skin thermoneutrality (Fig. 3) (Luiz et al., 2019). How is it that thresholds *in vivo* so widely differ? One answer emerges from differential expression of voltage-gated potassium channels that determine the probability of action potential firing. Recording from identified cold-sensing cells in culture revealed that the thermal threshold of a given neuron is reciprocally determined by the functional levels of Trpm8 and a 4-AP-sensitive voltage-gated potassium current (Madrid et al., 2009). This excitability brake current, I_KD_, is a voltage-gated hyperpolarizing current that acts to suppress depolarization induced by the cold. The molecular substrate of this current is yet to be conclusively determined, however, its block by dendrotoxins points to heteromers of the Shaker-like K_V_1.1 and K_V_1.2 channels as probable candidates (Madrid et al., 2009). These findings have been replicated by an unbiased constellation pharmacology screen, which demonstrated enhanced dendrotoxin-sensitivity of high-threshold versus low-threshold cold-sensing neurons (Teichert et al., 2014). Consistent with its purported role as an excitability brake, K_V_1.2-containing channels have also been shown to blunt cold responses purportedly mediated by Trpa1 (Memon et al., 2017).

### 2.6 Peripheral coding of cold sensation

Hitherto single ion channel genes have proved fruitful as genetic markers to define the cellular code for cold sensation through ablating of specific subsets of neurons. However, the advent of single-cell RNA sequencing has allowed for unbiased classification of DRG neurons based on the expression of many thousands of unique transcripts, revealing unexpected complexity in the peripheral coding of cold (Usoskin et al., 2014). The latest study distinguishes three types of Trpm8-positive cold-sensing neuron: two putative C fibre and one putative Aδ fibre population (Zeisel et al., 2018). These chime conveniently with the three functional archetypes of cold-sensing neuron recently uncovered by *in vivo* calcium imaging of cooling-evoked activity in the trigeminal ganglion (Leijon et al., 2019, Yarmolinsky et al., 2016).

*In vivo* imaging studies are pivotal to investigating the global representation of cooling in the periphery. There does appear to be a specific unimodal ‘labelled line’ encompassing neurons that detect both mild and noxious cooling, as well as a group of polymodal cells activated by the cold (Chisholm et al., 2018, Emery et al., 2016, Leijon et al., 2019, Luiz et al., 2019, Wang et al., 2018, Yarmolinsky et al., 2016) Cold-sensing neurons typically exhibit graded responses to enhanced cooling, consistent with a population code where decreasing temperature is correlated with increasing number and strength of neuronal responses (Chisholm et al., 2018, Luiz et al., 2019, Yarmolinsky et al., 2016). One dorsal root ganglia imaging study proposed a contrasting combinatorial strategy where cold-sensing neurons are tuned to respond only within a certain band of temperatures, and it is the pattern of co-activated neurons that reflects the degree of cooling (Wang et al., 2018). Given the clear molecular heterogeneity among cold-sensing neurons, it may be that distinct coding strategies are operating simultaneously in different cells. Indeed, in the trigeminal ganglia individual neurons that followed either coding scheme could be observed (Leijon et al., 2019). How exactly a unique complement of ion channels endows each cold-sensing neuron with its coding characteristics remains elusive.

## 3 Pathophysiology of cold allodynia

The *International Association for the Study of Pain* defines pain as ‘an unpleasant sensory and emotional experience associated with actual or potential tissue damage, or described in terms of such damage (IASP, 1979). This definition stresses that pain is a complex experience molded by numerous psychological and social factors. Yet, given chronic pain can be prevented in humans by peripheral nerve block, ascending input from sensory neurons is crucial to driving the experience of pain in health and disease (Aguirre et al., 2012).

While there is a wealth of data on the ion channels and sensory neurons controlling cold-sensing in the healthy state, the role of these cells and molecules in cold allodynia remains unclear. Mouse models of neuropathic pain exhibiting hypersensitivity to cooling are useful tools for mechanistic investigation of cold allodynia *in vivo*, with most studies focused on peripheral nerve injury- and chemotherapy-induced neuropathy (Jaggi et al., 2011). Although these studies are hampered by the challenges involved in assessing cold-evoked pain in mice, a number of molecules have been implicated in cold allodynia through behavioural experiments on KO and cell ablated animals (Table 2) (Deuis et al., 2017).

**Table 2:** Cold allodynia phenotype of ion channel KO and cell ablated mice. The symbol ‘↓’ denotes reduced or absent cold allodynia, ‘↑’ denotes enhanced cold allodynia and ‘–’ denotes unchanged cold allodynia in different mouse models of neuropathic pain following genetic manipulation of ion channels and sensory neuron subsets.

Mouse Line			Disease Model			
Type	Channels (s)	Manipulation	Complete Freud’s Adjuvant	Chronic Constriction Injury	Oxaliplatin	Ciguatera
Trp	*Trpm8*	KO	↓(Colburn et al., 2007, Knowlton et al., 2013)	↓(Colburn et al., 2007, Knowlton et al., 2013)	↓(Descoeur et al., 2011), –(Deuis et al., 2013)	–(Vetter et al., 2012)
		DTR	↓(Knowlton et al., 2013)			
	*Trpa1*	KO			↓(Nassini et al., 2011), –(Deuis et al., 2013)	↓(Vetter et al., 2012)

K2P	*TRAAK*	KO		– (Noël et al., 2009)		
	*TREK1*	KO		↓(Alloui et al., 2006)		
	*TRAAK/TREK1*	Double KO		↑(Noël et al., 2009)	↓(Descoeur et al., 2011)	
	*TREK2*	KO			↓(Pereira et al., 2014)	
	*TRAAK/TREK1/TREK2*	Triple KO			↓(Pereira et al., 2014)	
	*TRESK*	KO			↓(Castellanos et al., 2019)	

Na_V_	*Na_V_1.7*	Avil-Cre KO		↓(Minett et al., 2014a, Minett et al., 2014b, Minett et al., 2012)	–(Minett et al., 2014a, Minett et al., 2014b)	
		Wnt1-Cre KO		↓(Minett et al., 2014a, Minett et al., 2014b)	–(Minett et al., 2014a, Minett et al., 2014b)	
	*Na_V_1.8*	KO		↓(Leo et al., 2010, Minett et al., 2014a, Minett et al., 2014b)	–(Deuis et al., 2013, Minett et al., 2014a, Minett et al., 2014b)	↓(Vetter et al., 2012)
		DTA			–(Minett et al., 2014a, Minett et al., 2014b)	↓(Vetter et al., 2012)
	*Na_V_1.9*	KO		↓(Leo et al., 2010, Minett et al., 2014a, Minett et al., 2014b)	↓(Lolignier et al., 2015), –(Deuis et al., 2013, Minett et al., 2014a, Minett et al., 2014b)	–(Vetter et al., 2012)

### 3.1 Molecular mechanisms of cold allodynia

Although different chronic pain conditions may present with similar cold-evoked pain symptoms, the underlying disease mechanisms driving cold allodynia are often distinct. This is evident even in the mouse KO literature, where studies exist that either support or conflict with a role for a given channel or cell type in cold allodynia. Both Trpm8 KO and Trpm8-DTR mice show deficits in cold allodynia monitored by acetone responses after chronic constriction injury (Colburn et al., 2007, Pogorzala et al., 2013). Likewise, cold allodynia evoked by artemin, NGF, or morphine-induced hyperalgesia is dependent on the Trpm8 channel (Gong and Jasmin, 2017, Lippoldt et al., 2013). On the other hand, Trpm8 deletion has no effect on cold allodynia in a mouse model of ciguatera poisoning. Ciguatoxin-evoked cold hypersensitivity depends instead on Trpa1, corroborated by the emergence in culture of Trpa1-dependent cold responses in a set of normally cold-insensitive neurons (Vetter et al., 2012). Interestingly, Trpa1 and Trpm8 were both dispensable for acute cold allodynia evoked by a single intraplantar injection of oxaliplatin, reaffirming that alternative transducers mediating pathological cold pain must exist (Deuis et al., 2013). This is in contrast to systemic models of oxaliplatin where both KOs do apparently show deficits (Descoeur et al., 2011, Nassini et al., 2011).

Which sodium channel isoforms are required for afferent excitability in cold allodynia is equally controversial. Na_V_1.9 is absolutely required for cold allodynia induced by a single intraperitoneal injection of the chemotherapeutic oxaliplatin (Lolignier et al., 2015). On the other hand, Na_V_1.9 deletion did not affect cold allodynia in the intraplantar oxaliplatin model of cold allodynia; rather, Na_V_1.6 was shown to be essential through pharmacological block (Deuis et al., 2013). This fits with findings that oxaliplatin increases persistent and resurgent Na_V_1.6 currents in large diameter DRG neurons in culture, accompanied by increased spiking during cooling of A fibres from human nerve fascicles (Sittl et al., 2012). Given these divergent findings about the role of sodium channels in oxaliplatin neuropathy, it is clear that even within a chronic pain model with a well-defined etiology, the method of administration, time course and behavioural tests used can all impact the manifestation of gene KO phenotypes, pointing to mechanistic redundancy at the molecular level.

Chronic oxaliplatin causes substantial alterations in the expression of many ion channels, including numerous K_V_, K2P and HCN channels, all of which might contribute to cold allodynia (Descoeur et al., 2011). For example, the oxaliplatin-induced downregulation of K_V_1.1 mRNA agrees with reports that the brake current mediated by this channel is decreased in sensory neurons after chronic constriction injury. Block of this channel in mice is sufficient to cause behavioural cold hypersensitivity and blunts the manifestation of cold allodynia, identifying it as a pathway engaged in cold allodynia (González et al., 2017). Mouse K2P channel KOs are also basally hypersensitive to cooling, and do not develop additional cold allodynia in neuropathy (Castellanos et al., 2019). Finally, HCN channels – which control action potential firing – were shown to contribute to cold allodynia in chronic constriction injury by conditional deletion of HCN2 in Na_V_1.8-positive neurons (Emery et al., 2011).

Given the clear mechanistic link between cold-sensitivity and the excitability state of a neuron, a parsimonious explanation for the number of ion channels shown to contribute to cold allodynia may be that cold hypersensitivity is a product of general increases in terminal excitability, rather than dependent on a single transducer protein. Mutually-redundant and disease model-specific molecular changes can cause the cell to enter a hyperexcitable state. Any ion channel manipulation that dampens cell excitability might be sufficient to decrease firing to cooling in the neurons subserving cold allodynia, even if that particular molecule is not itself dysregulated by disease. In this case, gene KO experiments become particularly difficult to interpret, due to compensatory changes in ion channel expression (Akopian et al., 1999).

### 3.2 Cellular mechanisms of cold allodynia

Given the limitations of gene deletion experiments, investigation of transgenic mice where subsets of neurons are genetically ablated has been instructive in defining the cellular basis of cold allodynia. In the periphery, cold allodynia and tactile allodynia appear to require distinct ‘labelled lines.’ Ablation of all Trpv1 lineage neurons prevents cold, but not mechanical, allodynia in the spared nerve injury model (Cobos et al., 2018). In contrast, ablation of TrkB-positive cells in adulthood suggests these neurons are absolutely required for dynamic, punctate and static tactile allodynia, but dispensable for cold allodynia in the same model (Dhandapani et al., 2018). The Trpv1 lineage encompasses diverse types of sensory neuron, however, including cells corresponding to nociceptors and thermosensors, both C and A fibres (Cavanaugh et al., 2011).

Three major mechanisms have been proposed to explain how different subsets of sensory neurons drive cold allodynia. In the first mechanism, cold-activated nociceptors that normally respond to extreme cold can become responsive to milder temperature drops and consequently drive cooling-evoked pain. Calcium imaging of dissociated sensory neurons found altered activation thresholds of cold-sensitive cells may be responsible for cold allodynia (Castellanos et al., 2019, González et al., 2017). An *in vivo* imaging study which examined cold activity after ultraviolet burn injury did find greater activity with smaller temperature drops, suggesting that threshold shifts may contribute to this form of cold allodynia (Chisholm et al., 2018). This change in threshold has been attributed to functional downregulation of voltage-gated potassium channels, which normally act to limit neuron depolarization in the cold (Pertusa and Madrid, 2017).

In the second mechanism, cold-insensitive nociceptors that normally provide noxious sensory input acquire a *de novo* sensitivity to cooling. A recent *in vivo* imaging study of trigeminal ganglia showed that, following burn injury of the oral cavity, previously ‘silent’ neurons became newly sensitive to cooling (Yarmolinsky et al., 2016). These neurons were identified as peptidergic nociceptors on the basis of post-hoc immunohistochemical labelling for CGRPα. Tellingly, spared nerve injury-evoked cold allodynia can be temporarily reversed by optogenetic silencing of CGRPα-positive afferents expressing the inhibitory opsin archaerhodopsin-3, hinting at a contribution of these cells to cold allodynia (Cowie et al., 2018). That nociceptors are recruited to become cold-sensitive during chronic pain is supported by a microneurography study of a human patient with idiopathic small fibre neuropathy whose C-fibre nociceptors show aberrant cold-sensitivity associated with cold allodynia (Serra et al., 2009).

In the third mechanism, nociceptive input is unchanged and sensitization to innocuous cold input occurs centrally (Woolf, 1983). According to this hypothesis, afferent activity in response to cooling is normal and drives pain through aberrant spinal cord and brain circuits. A role for non-nociceptive cold neurons is supported by mouse experiments where quaternary lidocaine derivative QX-314 is targeted to silence only Trpm8-expressing neurons (Ongun et al., 2018). In humans, preferential blockade of large fibres abolishes cold allodynia in both non-freezing cold injury and oxaliplatin neuropathy, demonstrating a requirement for A fibres typically categorized as non-nociceptive (Forstenpointner et al., 2018, Jørum and Opstad, 2019). The three mechanisms outlined here are not mutually exclusive and may act in concert to drive cold allodynia.

### 3.3 Analgesic treatment of cold allodynia

What is the outlook for treating cold pain? Analgesic treatment of cold allodynia is typically symptomatic. For neuropathic pain patients, cold allodynia is one sensory abnormality among many, and may not even be the primary complaint. Despite advances in defining the mechanistic basis of cold allodynia, patients are treated using standard analgesics that do not rationally target specific pain modalities. This is compounded by clinical trials of analgesics where pain is assessed in a manner blind to modality using patient self-report rather than using quantitative sensory testing which could discriminate cold pain effects (Yin et al., 2015).

Given the vast number of molecules implicated in cold allodynia, rational therapy must be informed by a detailed understanding of what drives cold allodynia in that particular pain condition in that specific patient. As such personalized medicine currently lies out of reach, a promising alternative is to identify core pathophysiology that is agnostic to the particular molecular changes involved and that is in principle druggable. We have suggested that altered cell excitability due to dysregulated sodium and potassium channel conductances is a feature across multiple disease models of cold allodynia. An attractive strategy would be to reduce afferent terminal excitability. For instance, the voltage-gated potassium channel activators retigabine and flupirtine have been shown to reduce oxaliplatin induced-cold allodynia, presumably by shifting hyperexcitable afferents into a quiescent state through increased potassium efflux (Deuis et al., 2014). Clinical trials that include diabetic neuropathy patients suffering from cold pain show efficacy for sodium channel inhibitors (Eisenberg et al., 2001). Unfortunately, ensuring selectivity of such drugs remains a challenge. In preclinical studies, a chemogenetic strategy which selectively silences sensory neurons has been used to treat neuropathic cold allodynia, foreshadowing a potential gene therapy for patients with extreme cold pain (Weir et al., 2017). Such an approach would of course require a detailed knowledge of – and a means to target – sensory afferent subsets causally involved in cold allodynia in different chronic pain conditions.

## 4 Conclusion

**Fig. 4:**
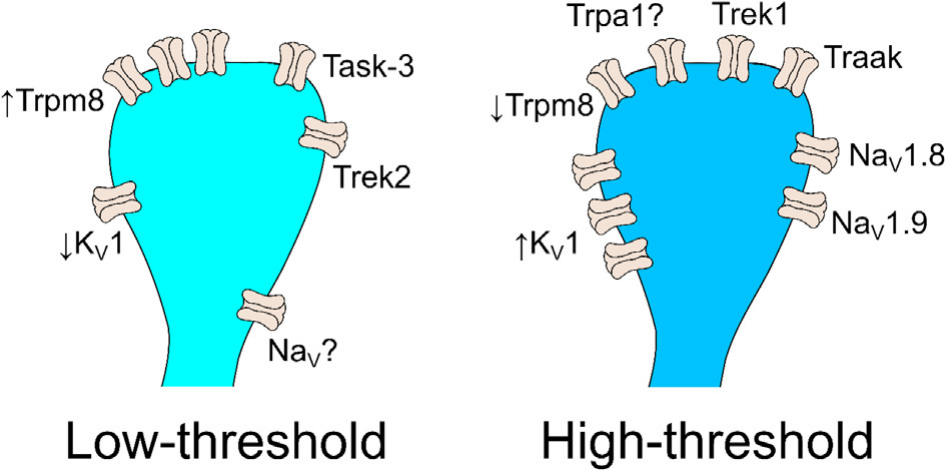
Ion channels defining low- and high-threshold cold-sensing neurons. Schematic illustrating the ion channels expressed in cold-sensing neurons that transduce cooling and control terminal excitability at low temperatures.

Cold-sensing neurons signal decreases in ambient or local temperature to guide homeostatic regulation, adaptive behaviour and subjective experience. The expression of unique constellations of ion channels tunes these neurons to respond to mild or extreme cooling (Fig. 4). Although Trpm8 has been established as the major detector of mild cooling in mammals, numerous other Trp and background potassium channels contribute to the initial transduction of cooling into afferent terminal depolarization. Whether the neuron in turn fires an action potential is dependent on voltage-gated sodium and potassium channels that govern afferent excitability. Ion channel dysfunction or aberrant expression that shifts membrane excitability can profoundly alter the cold-sensitivity of sensory neurons. In chronic pain patients suffering from cold allodynia, normally innocuous cooling elicits intolerable pain due to changes in the ion channel profile of sensory neurons that endows them with an increased or inappropriate sensitivity to cold. In the future, understanding which ion channels are dysregulated and in which sensory neurons will aid the development of rational analgesics for cold allodynia by targeting underlying pathophysiological mechanisms. Although our mechanistic understanding of cold-sensing in the healthy state is now relatively mature, we believe the cells and molecules driving cold allodynia remain ripe for discovery.
